# The Story of the Insane from Year to Year

**Published:** 1896-07-04

**Authors:** 


### THE STORY OF THE INSANE FROM YEAR TO YEAR.

Ninth Series.

### WONFORD HOUSE, EXETER.

This hospital for the insane contained on January 1st, 1895, 119 patients, and at the end of the year there were 123 in residence. There were during the year 29 admissions, 11 recoveries, 10 discharged relieved, and 3 deaths. The recoveries stand at 42·3 per cent. of the admissions, and the deaths at 2·4 on the average number resident. Hereditary tendency accounts for 11 of the admissions, overwork for 8, and drink for 4. Dr. Deas takes, in our opinion, a very sensible view of the system of voluntary boarders in our asylums. He thinks there is only a small margin "between the very mild cases of nervous disturbance, to whom the surroundings and atmosphere of an asylum are more likely to be injurious than beneficial, and those cases which are practically insane and can only be retained as boarders by straining the law more or less." No doubt this is so; but there is an opposite to the question, inasmuch as the reception of voluntary boarders must always have some little effect in disabusing the public mind of its extraordinary ideas about asylums. The commissioners continue to recommend the erection of a recreation-room, and Dr. Deas thinks the commissioners have not treated the asylum "considerately on this question." Dr. Deas quotes paragraphs from the reports, and adds that anyone reading them "might be excused for coming to the conclusion that Wonford House was in the Dark Ages and that no associated entertainments took place." We doubt whether anyone who knows Dr. Deas or Wonford House would come to that conclusion, for the asylum is well known to be admirably managed; but it is as well for the public to learn that there are two large rooms set apart for amusements, and that entertainments take place very often. Dr. Deas says, and we quite agree with him, that to have social evenings in a large recreation hall would be a mistake, as it "would tend to destroy their pleasant homely character." The average payments per week per patient has increased a little during the year, but so has the cost of maintenance. We are glad to note that five patients are received free of all charge; that ten are kept for less than 20s. a week, and one-half of the whole number are assisted to some extent. Dr. Deas was absent part of the year owing to ill-health, and we congratulate him on his recovery, in which congratulation everyone who knows Dr. Deas will join heartily.

### CORNWALL COUNTY ASYLUM.

This asylum contained 725 patients on January 1st, and that number had risen to 760 on December 31st. There were 146 admissions, 43 recoveries, and 54 deaths. The recoveries stand at 31·3 per cent. on the admissions, and the deaths at 7·1 on the average number resident. Cerebral and spinal diseases caused death in 20 cases, and consumption in 7. Hereditary influence caused the insanity in 14 of the admissions, intemperance in 7, and domestic trouble in 6. Dr. Adams says he found mechanical restraint necessary in a case of melancholia, with attempts at self-injury, and he rightly remarks that it was the most humane method of treatment. There can be no doubt that such restraint is in every way better than the employment of two or three attendants. No doubt it may be abused; but the same may be said of every form of treatment. Although the population of Cornwall is a decreasing one, the number of the registered insane is an ever-increasing one, and Dr. Adams points out that this is caused by legislation, and not by an actual increase of insanity. First we had the Common Chargeability Act of 1861; then the Act of 1873, which gave a capitation grant for every patient in an asylum; and finally we had the Lunacy Act of 1890. The experience of almost every county in

England is similar to that of Cornwall, and the pecuniary loss entailed on the ratepayers by these Acts must have been literally enormous. In the meantime asylums are overcrowded, and the only remedy at hand is to build. Naturally committees dislike this course, and yet delay necessarily means more and more outlay. The average weekly cost per head was 10s. 4d. for 1894.

### CUMBERLAND ASYLUM.

On January 1st this asylum contained 580 patients, and by the end of the year the number had risen to 591, while the average number resident was 594. There were 192 admissions, 85 recoveries, 34 discharged relieved, and 54 deaths. Calculated on the admissions the recoveries stand at 44·3 per cent., and the deaths at 9 per cent., calculated on the average number resident. Of the deaths, 12 were caused by cerebral or spinal diseases, and the same number by consumption. Of the admissions, 61 were owing to hereditary tendency, 15 to drink, and 9 to domestic trouble. Religion figures as the cause in 7, and love in 4. Dr. Campbell's remarks on the maintenance of private patients in county asylums are extremely good, and we regret being unable to quote them in full. "The royal asylums in Scotland," he says, "have for years provided suitable accommodation for private patients at moderate rates of board, and this to a certain extent accounts for the higher proportion of privately maintained insane. England has not been hitherto so well situated in this respect; but when accommodation for private patients is provided in connection with known public institutions we shall probably see an increase in the number of private patients, and a decrease in the rate-supported." We believe this view to be correct, so that in providing blocks for private patients asylum committees would make money on one hand, and save it on the other. The visitors say "they consider that the efforts made by Dr. Campbell and the staff of officials are well evidenced by the satisfactory recovery rate and low death rate." The average weekly cost per head is 8s. 8d.

